# Evaluation of sampling and quenching procedures for the analysis of intracellular metabolites in CHO suspension cells

**DOI:** 10.1186/1753-6561-5-S8-P82

**Published:** 2011-11-22

**Authors:** Judith Wahrheit, Jens Niklas, Elmar Heinzle

**Affiliations:** 1 Biochemical Engineering Institute, Saarland University, 66123 Saarbrücken, Germany

## Background

Metabolomics, aiming at the quantification of all extracellular and intracellular metabolites, is a valuable tool for characterizing, understanding and manipulating the physiology of mammalian cells. While extracellular metabolite analysis is well established, required quenching and extraction procedures for intracellular metabolite analysis in mammalian suspension cells are not yet routinely available. In this study a simple sampling and quenching protocol using ice-cold 0.9% saline as quenching solution [1] was tested on CHO cells. Quenching efficiency, preservation of cell integrity as well as cell separation and the necessity of washing steps were evaluated and possible sources of error are discussed.

## Materials and methods

The antithrombin-III producing CHO cell line T-CHO-ATIII was cultivated in serum free CHO-S-SFM II medium (Gibco/Invitrogen, Carlsbad, CA, USA). Cells were kept in baffled shake flasks (Corning, Corning, NY, USA) in a shaking incubator (2 in. orbit, Innova 4230, New Brunswick Scientific, Edison, NJ, USA) at 37°C with a constant 5% (v/v) CO_2_ supply at 185 rpm. Quenching was performed by mixing 5 ml of cell suspension with 45 ml ice-cold 0.9% ice-cold saline as quenching solution (QS) [1]. Unless otherwise stated, sampling was done by centrifuging for 1 min at 1000g followed by shock-freezing of cell pellets in a CO_2_-acetone bath. Intracellular metabolites were extracted from freeze-dried cell pellets using ice-cold 50% acetonitrile [1]. Survival of the cells in the QS at 0°C was tested after sampling at different relative centrifugal forces (RCF, 1000g – 2000g – 3000g – 4000g). Cell number, viability and cell morphology of cells re-suspended in QS were checked during incubation at 0°C using an automated cell counter (Countess, Invitrogen, Karlsruhe, Germany). Carryover of extracellular metabolites from the culture medium was investigated without washing and after applying different washing strategies. Cell pellets were either re-suspended in 50 ml QS or rinsed with 50 ml QS followed by another centrifugation step. Cell numbers and extracellular metabolites were analysed and compared to the initial sample. Quenching efficiency at 0°C was investigated by measuring enzyme activity and by monitoring intracellular metabolite amounts at 0°C. Lactate dehydrogenase activity was determined in quenched cells (0°C) and in cells without quenching (37°C) after cell permeabilization with 0.05% Triton-X-100 [2]. Intracellular citrate, α-ketoglutarate, pyruvate, succinate, lactate and fumarate were quantified in cell extracts after incubation of quenched cell suspensions at 0°C for 0, 5 or 10 min.

## Results

Cell integrity was maintained in ice-cold 0.9% saline for at least 30 min. Centrifugation at 1000g for 1 min led to 50% cell loss during sampling. Sampling at higher RCF increased cell yield to 65%. However, sampling at RCF higher than 2000g did not further increase cell yield but seemed to affect cell viability. Unchanging cell diameter and morphology before and after quenching and during incubation at 0°C indicated that no osmotic stress and no biased selection of cells during centrifugation occurred. Contamination with culture medium was found relatively low even without any washing steps. Less than 0.25% pyruvate and lactate and less than 0.1% glucose compared to the amounts measured in the extracellular medium of the cell suspension were found after quenching. Citrate found in the initial sample was not detected after quenching in any of the samples with or without washing. Furthermore, after washing by rinsing or re-suspending the cell pellets no glucose and less than 0.2% pyruvate and lactate could be detected. Washing by resuspending did not yield better results than rinsing the cell pellet but can possibly lead to cell damage and leakage of intracellular metabolites as indicated by traces of fumarate detected in the sample. The inclusion of washing steps further decreases cell yield. Cell loss after rinsing the cell pellet was very low (48% cell recovery compared to 56% without washing). In contrast, washing by re-suspending the pellet led to a tremendous cell loss; only 19% of the initial cell number could be recovered. In quenched cells enzyme activity was efficiently stopped as shown by comparing lactate dehydrogenase activity in quenched cells (specific activity 0.33 ± 1.1 pmol/(cell×h)) and control cells kept at 37°C (15.17 ± 0.19 pmol/(cell×h)). Increasing concentrations of pyruvate, lactate and fumarate after 10 min incubation of quenched cell suspensions at 0°C indicate that metabolism is not completely stopped at 0°C. However, metabolic activity seemed to be sufficiently slowed down within the first 5 min of incubation where no significant change of intracellular metabolites was observed.

## Conclusions

Ice-cold 0.9% saline proved to be a suitable QS maintaining cell integrity and cell morphology. Sampling via centrifugation at RCF higher than 2000g seemed to affect cell viability and should be avoided. By using a tenfold excess of QS compared to the sample volume rapid cooling of the sample could be achieved and contamination with culture medium was found relatively low even without any washing steps. Carryover of extracellular metabolites can be further reduced by rinsing the cell pellet with an excess of QS. Washing by re-suspending the cell pellet diminished cell yield tremendously and can possibly lead to cell damage resulting in leakage of intracellular metabolites. It is therefore not suitable. In quenched cells enzyme activity was efficiently halted within the first 5 min of incubation at 0°C indicating sufficient quenching of metabolic activity for the analysis of intracellular metabolites with lower turnover. Sampling and washing should therefore be completed within the first 5 min after quenching.
